# Single-cell analysis of differences in transcriptomic profiles of oocytes and cumulus cells at GV, MI, MII stages from PCOS patients

**DOI:** 10.1038/srep39638

**Published:** 2016-12-22

**Authors:** Qiwei Liu, Yumei Li, Yun Feng, Chaojie Liu, Jieliang Ma, Yifei Li, Huifen Xiang, Yazhong Ji, Yunxia Cao, Xiaowen Tong, Zhigang Xue

**Affiliations:** 1 Department of Gynecology and Obstetrics, Affiliated Tongji Hospital, Tongji University, Shanghai, China; 2 Reproductive Medical Center of XiangYa Hospital, Affiliated Central South University, Changsha, China; 3 Translational Center for Stem Cell Research, Tongji Hospital, Department of Regenerative Medicine, Tongji University, Shanghai, China; 4 Reproductive Medicine Center, The First Affiliated Hospital of Anhui Medical University, Hefei, China; 5 Reproductive Medicine Center, Affiliated Tongji Hospital, Tongji University, Shanghai, China

## Abstract

Polycystic ovary syndrome (PCOS) is a common frequent endocrine disorder among women of reproductive age. Although assisted reproductive techniques (ARTs) are used to address subfertility in PCOS women, their effectiveness is not clear. Our aim was to compare transcriptomic profiles of oocytes and cumulus cells (CCs) between women with and without PCOS, and assess the effectiveness of ARTs in treating PCOS patients. We collected oocytes and CCs from 16 patients with and without PCOS patients to categorize them into 6 groups according to oocyte nuclear maturation. Transcriptional gene expression of oocyte and CCs was determined via single-cell RNA sequencing. The ratio of fertilization and cleavage was higher in PCOS patients than in non-PCOS patients undergoing ARTs, and there was no difference in the number of high-quality embryos between the groups. Differentially expressed genes including *PPP2R1A, PDGFRA, EGFR, GJA1, PTGS2, TNFAIP6, TGF-β1, CAV1, INHBB et al.* were investigated as potential causes of PCOS oocytes and CCs disorder at early stages, but their expression returned to the normal level at the metaphase II (MII) stage via ARTs. In conclusion, ARTs can improve the quality of cumulus-oocyte complex (COC) and increase the ratio of fertilization and cleavage in PCOS women.

Polycystic ovary syndrome (PCOS) is one of the most frequent endocrine disorders in women of reproductive age, characterized by hyperandrogenemia, oligo-ovulation or anovulation, and polycystic ovaries^1^. PCOS affects 5–20% of reproductive women and causes subfertility in 50% of them^2^. Various methods are used to address subfertility in this population, including lifestyle modifications, ovulation induction, laparoscopic ovarian drilling, and assisted reproductive techniques (ARTs).

ARTs are increasingly used as primary treatment; however, whether ARTs improve oocyte quality and further results such as fertilization, cleavage, embryo and pregnancy in PCOS patients has long been controversial. Some studies have shown that ARTs can improve fertilization, cleavage, implantation, clinical pregnancy, and live-birth rates in women with PCOS, and patients with PCOS had similar or better pregnancy rates when comparing with control^3^,^4^,^5^. Wang *et al*. compared clinic outcomes of 2632 PCOS women with 28523 non-PCOS women undergoing ARTs, and found that there are higher number of available embryo and clinic pregnancy rate in PCOS women than in non-PCOS^6^. A meta-analysis showed that birth and implantation rates were higher in PCOS patients than in controls using *in vitro* maturation^7^. Heijnen *et al*. also reported PCOS and control patients undergoing conventional *in vitro* fertilization had similar pregnancy and live birth rates^8^. But others have been concerned that ARTs may decrease the quality of oocytes and delay the mutation process of oocytes in PCOS patients^9^,^10^,^11^,^12^. Previous studies used microarrays to analyze genes that are differentially expressed in cumulus cells (CCs) between PCOS patients and control patients. *EREG, AREG, ERK1/2, MAP3K4, LHCGR* and *TNIK*, among others, were related to the development of CCs in PCOS patients, but the complex genetic etiology of PCOS at different phases of oocyte and CCs development via ARTs is not yet clear^13^,^14^,^15^,^16^.

Therefore, we sought to analyze cumulus-oocyte complexes (COCs) containing oocytes and CCs at various stages, including germinal vesicle (GV, oocyte maturation arrested in the prophase of meiosis), metaphase I (MI, first meiotic metaphase), and metaphase II (MII, second meiotic metaphase). COCs are vital part of follicular phase, and their developmental process determines the quality of oocytes and enhances ratio of fertilization^17^. Furthermore, we used single-cell RNA sequencing (RNA-seq) technology to analyze transcriptome levels among COCs from PCOS and control patients.

The aim of this study was to compare the development of COCs between women with and without PCOS undergoing ARTs, which can help us assess the impact of ARTs on fertility in PCOS patients. The results may inform the appropriate use of ARTs in subfertile PCOS patients in clinical treatment.

## Results

### Clinical and biochemical characteristics of PCOS and control subjects

The clinical and biochemical characteristics of 42 non-PCOS patients and 42 PCOS patients who participated in this study are shown in Table 1. There was statistically significant difference between the two groups in age, body mass index (BMI), luteinizing hormone (LH), follicle-stimulating hormone (FSH), systolic blood pressure, diastolic blood pressure, insulin, total gonadotropin dose, androstenedione, sex hormone-binding globulin, mature oocytes, or high-quality embryos. Patients with PCOS had a higher ratio of basic LH/FSH (1.4 ± 0.9) and LH (9.3 ± 6.1) than control patients, LH/FSH (0.72 ± 0.30) and LH (4.8 ± 2.5), P < 0.001. The mean number of antral follicles in the PCOS patients (>24) was significantly higher than that in the non-PCOS patients (13 ± 5). Mean testosterone levels, total ovarian volume, the mean number of aspirated oocytes, and the ratio of oosperm and cleavage were significantly higher in the PCOS group (5475.9 ± 1800.8, 20, 80.1 ± 17.2, 79.4 ± 18.6, respectively) than in the non-PCOS group (3737 ± 1863.7, 14, 71.4 ± 21.7, 70.0 ± 21.7, respectively).

### Profiles of differentially expressed genes in human CCs and oocytes

**Figure 1:**
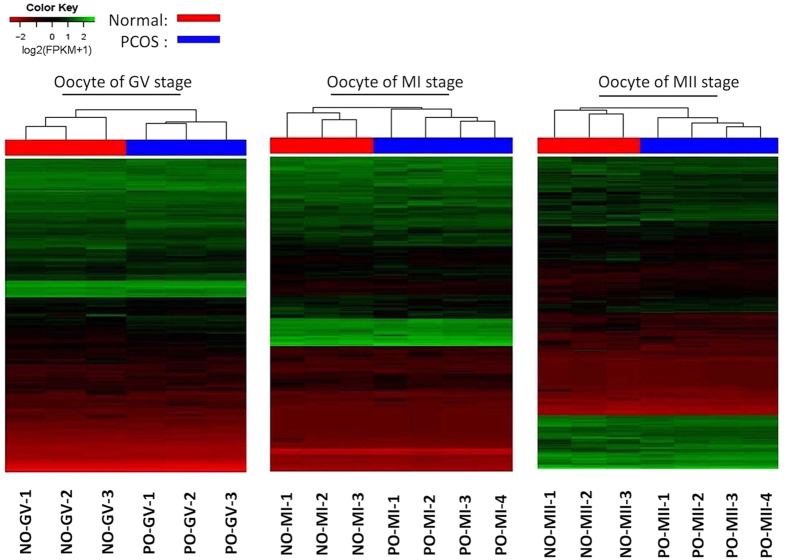
Heatmap of differentially expressed genes in oocytes with and without PCOS at GV, MI, MII stage. Green corresponds to up-expressed genes; red corresponds to down-expressed genes. NO-GV, oocyte at GV stage from normal women; PO-GV, oocyte at GV stage from women with PCOS; NO-MI, oocyte at MI stage from normal women; PO-MI, oocyte at MI stage from women with PCOS; NO-MII, oocyte at MII stage from normal women; PO-MII, oocyte at MII stage from women with PCOS.

**Figure 2:**
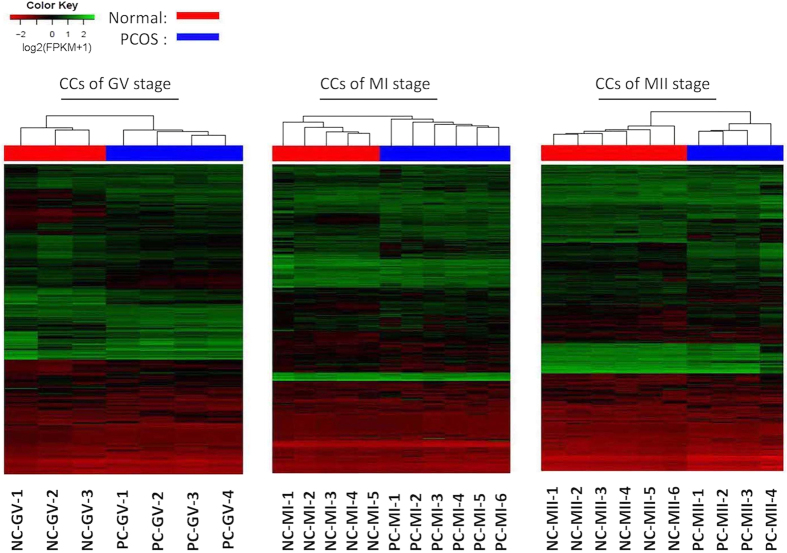
Heatmap of differentially expressed genes in CCs with and without PCOS at GV, MI, MII stage. Green corresponds to up-expressed genes; red corresponds to down-expressed genes. CCs, cumulus cells. NC-GV, CCs at GV stage from normal women; PC-GV, CCs at GV stage from women with PCOS; NC-MI, CCs at MI stage from normal women; PC-MI, CCs at MI stage from women with PCOS; NC-MII, CCs at MII stage from normal women; PC-MII, CCs at MII stage from women with PCOS.

Unsupervised clustering showed differential CC and oocyte gene expression between PCOS patients and controls (Figs 1 and 2). In class I (PCOS oocytes versus normal oocytes at the GV stage), 156 genes were upregulated (fold change [FC], from 3 to 8), and 450 genes were downregulated (FC, from -3 to -7). In class II (PCOS oocytes versus normal oocytes at the MI stage), 184 genes were upregulated (FC, from 2 to 5), and 253 genes were downregulated (FC, from -2 to -6). Last, in class III (PCOS oocytes versus normal oocytes at the MII stage), 487 genes were upregulated (FC, from 3 to 7), and 352 genes were downregulated (FC, from -3 to -8). When CCs were compared between the PCOS and non-PCOS groups, 4371, 1587, and 2450 upregulated genes, as well as 985, 780, and 1504 downregulated genes were found in class IV (PCOS CCs versus normal CCs at the GV stage), class V (PCOS CCs versus normal CCs at the MI stage), and class VI (PCOS CCs versus normal CCs at the MII stage), respectively (FC > 3 or FC < −3) (Table 2).

### Signal transduction pathways in oocytes and CCs from women with and without PCOS

**Figure 3:**
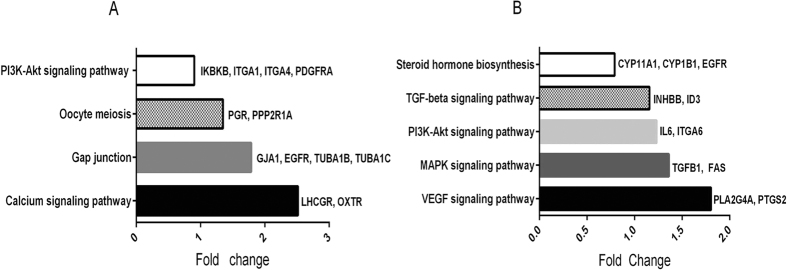
Significantly signaling pathway involved differentially expressed genes. The threshold of *P*adj was set at < 0.05 to find significantly signaling pathway. The listed genes are the differentially expressed genes analyzed in our study in each pathway. (**A**) Significantly signaling pathway of oocytes comparing PCOS patients with non-PCOS patients. (**B**) Significantly signaling pathway of CCs comparing PCOS patients with non-PCOS patients.

The functional enrichment analysis based on data from the Kyoto Encyclopedia of Genes and Genomes (KEGG) revealed that differentially expressed genes in PCOS oocytes were mainly involved in the PI3K-Akt signaling pathway, gap junction pathway, calcium signaling pathway, and oocyte meiosis pathway (adjusted *P* value (*P*adj) < 0.05) (Fig. 3A), indicating those pathways may play important role in oocyte maturation. PI3K-Akt signaling pathway and gap junction pathway included 4 differential expressed genes in PCOS oocyte which were related to oocyte development respectively, while each calcium signaling pathway and oocyte meiosis pathway contained 2 differential expressed genes. In PCOS CCs, the differentially expressed genes were mainly involved in the MAPK signaling pathway, steroid hormone biosynthesis pathway, transforming growth factor, beta (TGF-β) signaling pathway, vascular endothelial growth factor (VEGF) signaling pathway, and PI3K-Akt signaling pathway (*P*adj < 0.05) (Fig. 3B). Briefly, we found 3 differentially expressed genes in steroid hormones pathway that are associated with COCs proliferation and maturation in PCOS CCs. We also found 2 differentially expressed genes in each of MAPK signaling pathway, TGF-β signaling pathway, VEGF signaling pathway, and PI3K-Akt signaling pathway.

### Differentially expressed genes in PCOS oocytes related to meiosis, gap junction, hormone receptor signaling, DNA repairing and secreted factors

**Figure 4:**
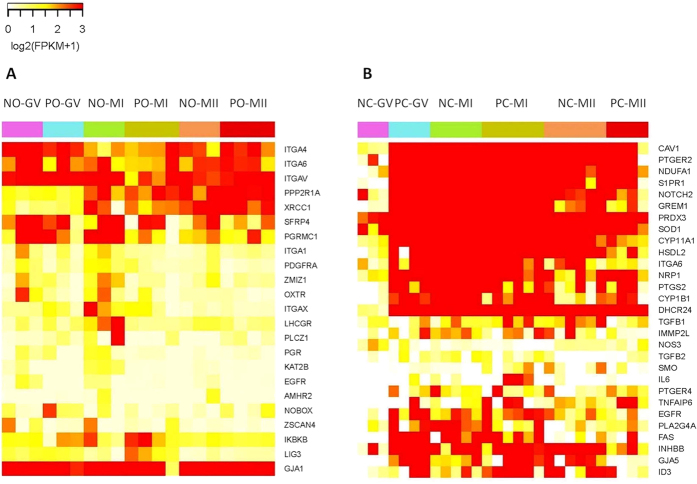
Heatmap of the expression patterns of selected genes at different stages. (**A**) Heatmap of expression patterns of selected genes in oocyte groups. (**B**) Heatmap of expression patterns of selected genes in CCs groups.

**Figure 5:**
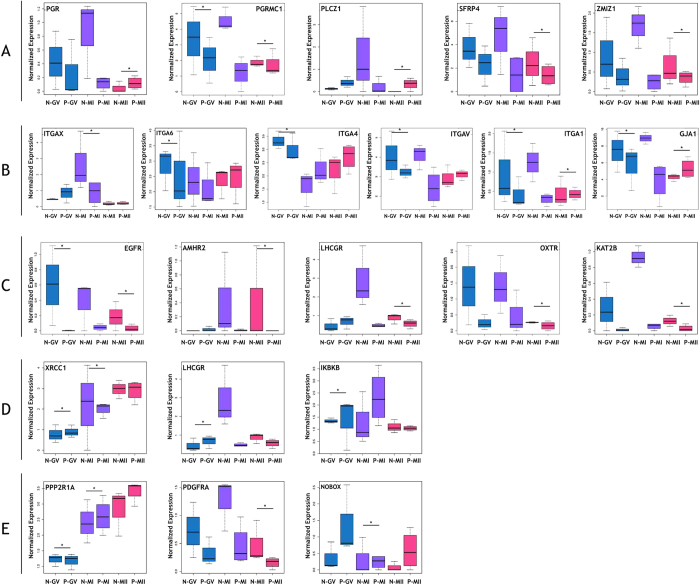
Differentially expressed genes in PCOS oocyte studied through single-cell RNA sequencing. The threshold of *P*adj was set at < 0.05 to find significantly expressed genes. (**A**) Boxplot showing differentially expressed genes involved in meiosis process. (**B**) Boxplot showing differentially expressed genes included in gap junction pathway. (**C**) Boxplot showing differentially expressed genes related to hormone receptor signaling. (**D**) Boxplot showing differentially expressed genes involved in DNA repairing. (**E**) Boxplot showing significantly expressed genes are related to secreted factors. *Padj < 0.05. The y axis indicates the log2 (FPKM + 1) expression levels.

Next, genes with significantly differential expression in PCOS oocytes compared with non-PCOS oocytes were analyzed (*P*adj < 0.05) (Fig. 4A). Many genes related with oocyte meiosis and maturation were differentially expressed in PCOS oocytes compared with non-PCOS oocytes, including epidermal growth factor receptor (*EGFR*), progesterone receptor (*PGR*), progesterone receptor membrane component 1 (*PGRMC1*), phospholipase C, zeta 1 (*PLCZ1*), secreted frizzled-related protein 4 (*SFRP4*), and zinc finger, MIZ-type containing 1 (*ZMIZ1*) and zinc finger and SCAN domain containing 4 (*ZSCAN4*) (Fig. 5A). These genes that stimulate the meiosis process and oocyte maturation were significantly downregulated at the GV stage in PCOS oocytes compared with control oocytes, but their expression was recovered and normal at the MI and MII stages. Several genes that regulate cell-cell communication were downregulated in PCOS CCs compared with control CCs, such as the integrin (ITG) family (subunits *ITGAX, ITGAV, ITGA1, ITGA4,* and *ITGA6*) and gap junction protein alpha 1 (*GJA1*/*CX43*) at three stages (Fig. 5B). Hormone receptors were differentially expressed in PCOS oocytes compared with non-PCOS oocytes at the mutational stage, including anti-Müllerian hormone receptor, type II (*AMHR2*), *LHCGR*, oxytocin receptor (*OXTR*) and K (lysine) acetyltransferase 2B (*KAT2B*) (Fig. 5C). The expression of genes related to DNA repair, including inhibitor of kappa light polypeptide gene enhancer in B-cells, kinase beta, ligase I, DNA, ATP-dependent, and X-ray repair complementing defective repair in Chinese hamster cells 1 (*XRCC1*), was higher in PCOS oocytes than in normal oocytes at early phases, but the expression of these genes was normal at the MII phase (Fig. 5D). Genes related to secreted factors, such as subunit alpha of protein phosphatase 2A (*PPP2R1A*), platelet-derived growth factor receptor, alpha polypeptide (*PDGFRA*), and *NOBOX* oogenesis homeobox (*NOBOX*) were differentially expressed; however, the expression of these secreted genes was normal at the maturation phase (Fig. 5E).

### Differentially expressed genes in PCOS CCs related to cell proliferation, hormone receptor signaling, folliculogenesis, gap junction, and oxidative stress

**Figure 6:**
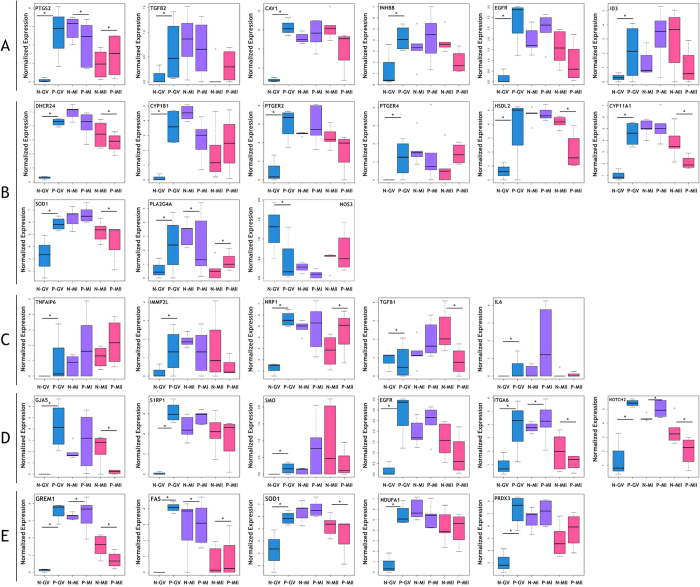
Differentially expressed genes in PCOS CCs studied through single-cell RNA sequencing. The threshold of *P*adj was set at < 0.05 to find significantly expressed genes. (**A**) Boxplot showing differentially expressed genes involved in CCs expansion. (**B**) Boxplot showing differentially expressed genes related to hormone receptor signaling. (**C**) Boxplot showing differentially expressed genes included in folliculogenesis. (**D**) Boxplot showing differentially expressed genes involved in gap junction. (**E**) Boxplot showing significantly expressed genes related to oxidative stress and genes predicting quality of oocyte. *Padj < 0.05. The y axis indicates the log2 (FPKM + 1) expression levels.

Genes with significantly differential expression in PCOS CCs when comparing with non-PCOS CCs were analyzed (Fig. 4B). Among many genes that are involved in cell proliferation, several genes were deregulated in PCOS CCs compared with non-PCOS CCs, including prostaglandin-endoperoxide synthase 2 (*PTGS2*), tumor necrosis factor-alpha-induced protein 6 (*TNFαIP6*), TGF-β2, caveolin 1 (*CAV1*), inhibin, beta B (*INHβB*), *EGFR*, and inhibitor of DNA binding 3 (*ID3*) (Fig. 6A). At the GV stage, all these genes were overexpressed in PCOS CCs, which suggests CCs proliferated in PCOS patients were overexpressed; however, there was no difference in expression at later stages. Genes related to hormone receptors, including phospholipase A2 group IVA (*PLA2G4A*); 24-dehydrocholesterol reductase (*DHCR24*); cytochrome P450, family 1, subfamily B, polypeptide 1 (*CYP1B1*); prostaglandin E receptor 2 (*PTGER2*); *PTGER4*; hydroxysteroid dehydrogenase like 2 (*HSDL2*); cytochrome P450, family 11, subfamily A, polypeptide 1 (*CYP11A1*); superoxide dismutase 1 (*SOD1*); and nitric oxide synthase 3 (*NOS3*), were significantly differentially expressed in PCOS patients compared with non-PCOS patients at baseline (Fig. 6B). And genes that impact the maturation of follicles and oocytes were also deregulated in PCOS CCs compared with non-PCOS CCs (Fig. 6C). These genes included *TNFαIP6*, inner mitochondrial membrane peptidase subunit 2 (*IMMP2L*), neuropilin 1 (*NRP1*), *TGF-β1*, and interleukin 6 (*IL6*). Several genes that regulate cell-cell communication were upregulated at the GV phase and normal or downregulated at the MI and MII stages in PCOS CCs compared with control CCs. These included gap junction protein, alpha 5 (*GJA5*), sphingosine-1-phosphate receptor 1 (*S1PR1*), Smoothened (*SMO*), *EGFR*, *ITGA6*, and *NOTCH2* (Fig. 6D). Many genes were reported can be prediction genes for the quality of embryo. In our study, Gremlin-1 (*GREM1*), fas cell surface death receptor (*FAS*), and superoxide dismutase 1 (*SOD1*), which have been shown to predict the quality of oocytes, were differentially expressed in PCOS CCs compared with control CCs. Last, genes related to oxidative stress, NADH dehydrogenase (ubiquinone) 1 alpha subcomplex, 1 (*NDUFA1*) and peroxiredoxin 3 (*PRDX3*), were differentially expressed in PCOS CCs compared with normal CCs (Fig. 6E).

In summary, genes that participate in vital processes such as cell proliferation, hormone receptors signaling, gap communication, folliculogenesis, and oxidative stress were all abnormally expressed in PCOS CCs compared with control CCs at the GV stage, but all of them were recovered to normal levels at the MII stage.

## Discussion

The purpose of our study was to use single-cell RNA-sequencing to illuminate differential expression of transcriptome in oocytes and CCs at the GV, MI, and MII stages in women with or without PCOS. Advances in RNA-sequencing technology are obvious compared with microarray methods, and the field of single-cell transcriptome analysis has developed quickly in recent years^18^,^19^,^20^. Traditional techniques may reflect only the average transcriptome of cells and may yield misleading results. In addition, we showed that our technique has greater sensitivity and coverage than microarray methods after analyzing three samples of individual morula embryos. Last, single-cell RNA-seq can detect heterogeneous single cells such as oocytes and CCs.

RNA-sequencing profiles suggested that genes in PCOS COCs mediated meiosis, hormone receptor signaling, cell-cell communication, proliferation, folliculogenesis, DNA repair, and oxidative stress were differentially expressed at an immature stage. These genes in oocytes were included in the PI3K-Akt signaling pathway, gap junction pathway, calcium signaling pathway, and oocyte meiosis pathway, and genes in CCs were included in the MAPK signaling pathway, steroid hormone biosynthesis pathway, TGF-β signaling pathway, VEGF signaling pathway, and PI3K-Akt signaling pathway. It was found that PI3K-Akt signaling pathway participates in COCs development in PCOS women because this signaling pathway is related to hyperandrogenism, insulin resistance, inflammation and oxidative stress^21^.

Connecting transcriptome profiles with clinical data, these results showed that genes involved in those important processes in PCOS CCs and oocytes were significantly differentially expressed at the GV stage, but with ARTs, the gene expression was comparable in mutational COCs (at the MII stage) between PCOS samples and control samples, which indicates that ARTs could help PCOS patients obtain relatively healthy oocytes at the transcriptome level, as well as a higher ratio of fertilization and cleavage.

Oocyte maturation is promoted by the resumption of meiosis, and inaccurate completion of this process may cause genetic infertility and pregnancy loss^22^. Recent findings indicated that FSH activates *EGFR*, which can induce oocyte meiosis resumption and maturation through the activation of MAPK3/1^23^. It suggested that *EGFR* could be used as gene marker of oocyte competence in mammals^24^. *PGR* and *PGRMC1* are nuclear transcription factors that initiate a signaling cascade during the maturation of COCs and are required for cumulus expansion and oocyte meiotic progression. The absence of these transcription factors can arrest oocyte maturation^25^,^26^. *PLCZ1*, which is significantly related to oocyte meiosis and the regulation of insulin secretion, can induce the activation of oocytes at the MII stage so that sperm and oocytes can fuse well^27^,^28^. The expression of *SFRP4* and *ZMIZ1*, which are regulators of ovulation and luteinization, is lower in PCOS oocytes, indicating that the abnormal microenvironment of PCOS impacts luteinization negatively^29^,^30^. *ZSCAN4,* a marker for the transition of RNA polymerase II-mediated transcription during GV oocyte maturation, is activated in GV oocyte^31^. In PCOS patients, *ZSCAN4* is only downregulated, at GV stage but not MII stage. All of those genes are downregulated in PCOS oocytes at an early stage (Fig. 5), suggesting that PCOS oocyte maturation was delayed at GV stage.

The *ITGA* gene family is integral transmembrane glycoproteins that connect cells and extracellular matrix, which can send information into cells. Therefore, one function of *ITGA* genes is the bridge of cell-cell interaction, cell adhesion, and signal transduction. A previous study demonstrated that *ITGA9* connects with *ADAM2* to mediate sperm-egg interactions and general cell adhesion^32^. In the current study, the expression of *ITGA* genes was significantly lower in PCOS oocytes than in normal oocytes, including *ITGA1, ITGA4, ITGA6, ITGAV,* and *ITGAX*. GJA1 (CX43), which localizes in gap junctions between oocyte and CCs and participates in primordial follicle assembly, is downregulated in PCOS oocytes, suggesting that oocytes in PCOS patients may not connect well with the surrounding CCs^33^. Overexpression of *AMHR2* increases the binding of AMH to it, which attenuates follicular or oocyte maturation^34^. Therefore, *AMHR2* is a possible indicator for the quality of embryo^35^. In PCOS oocytes, hormone receptors including *LHCGR, FSHR, AMHR2, KAT2B,* and *OXTR* were downregulated in the MII stage, which reflects the ability of oocyte to respond the higher hormone surge and protect the progress of oocyte mutation.

DNA repair pathways are more active in human immature oocytes when double-strand DNA breaks, which may defer meiotic resumption^36^. DNA-regulating genes such as *XRCC1, LIG* and *RAD54L* were overexpressed and activated to a higher degree in PCOS oocytes at the GV and MI stages, which can be detected DNA damaging was existed in PCOS women at early stages.

A previous study showed that depletion of *PPP2R1A* leads to an increase the activity of *CDK1* and cyclin B1, maturation promoting factor, which stimulates the transformation of the GV stage to the MI stage^37^. Therefore, *PPP2R1A* plays a negative role in oocyte meiotic maturation. In our study, PCOS oocytes had a higher expression of *PPP2R1A* than non-PCOS oocytes at the GV and MI stages, which suggested that maturation promoting factor was inhibited, thereby causing oocyte meiosis arrest at the GV and MI stages. *PDGFRA*, located in both oocytes and CCs, is a growth factor that positively influences early follicle growth^38^. The transcription factor *NOBOX* is essential for follicle formation and oocyte survival, and *NOBOX* regulates the expression of *GDF9* in humans, which is a secreted factor that is required for ovarian folliculogenesis^39^,^40^.

Summarily, PCOS oocytes have dysfunctional meiosis maturation, gap junction, hormone response, DNA damaging and secreted factors in the early phase. Dysfunction of these genes deferred oocyte meiosis at the GV stage and may inhibit fertilization and other processes. However, these problems can be solved via ARTs according to the expression levels at the MII stage and clinical outcomes.

*TNFαIP6*-deficient females are sterile, because this deficiency prevents CCs from assembling with their hyaluronan-rich extracellular matrix^41^. *TNFαIP6,* which affects cumulus expansion and oocyte maturation, was overexpressed in PCOS CCs, suggesting acceleration in the maturation of the CCs phenotype^13^. *PTGS2* increases the expansion of CCs and also plays an important role in the interaction of CCs with oocytes in the periovulatory period^42^. *TGF-β2* is a member of retinoblastoma protein and regulates the proliferation of CCs, and *CAV1* is regulated by LH, and its decreased expression is associated with the deregulation of granulosa cell differentiation^43^. In mice, downregulation of *INHβB* is shown to significantly increase apoptosis and inhibit steroidogenesis in granulosa cells^44^. And *INHβB* is overexpressed in PCOS CCs, which could have increased the proliferation and steroidogenesis of CCs. Overexpression of *EGFR* in CCs can enhance the proliferation of CCs and oocyte meiosis resumption and maturation through the activation of *MAPK3/1*^23^. ID3 is an AMH target gene and involved in the effects of AMH on the differentiation of CCs, and overexpression of the ID gene inhibits CCs expansion^45^.

Abnormal proliferation of PCOS granulose cells including CCs has been reported in many previous studies. However, the conclusions of these reports are varied. Several researchers demonstrated the increased proliferation of PCOS granulose cells compared with normal granulose cells, while others found no difference in the proliferation of granulose cells between PCOS and control samples^46^,^47^. In our study, the profiles indicated that genes responsible for the proliferation of PCOS CCS were upregulated at the GV stage, which resulted in more immature follicular than normal. But after ARTs, the expression of those genes was restored to its normal level.

*NOS* regulates LH to affect CCs. Higher expression of NOS can cause LH to stimulate the proliferation of CCs^48^. Recent research showed higher *SOD1* gene expression in CCs in infertile women, which could be a biomarker of clinical pregnancy after Intra-Cytoplasmic Sperm Injection (ICSI)^49^. Expression of the *PLA2G4A* gene is regulated by LH/hCG-dependent induction in granular cells via the adenylyl cyclase/cAMP pathway; therefore, lower expression of *PLA2G4A* in PCOS CCs reflected an abnormal LH surge^50^. The LH receptor *LHCGR* allows the development of preovulatory follicle and leads to ovulation. Lower expression of *LHCGR* in PCOS CCs may be a response to increased LH levels, oligomenorrhea, resistance to LH, infertility^51^. PTGER, the prostaglandin E (PGE) receptor, is expressed in oocytes and CCs. PGE-PTGER signaling regulates CCs expansion and reorganization, which is required for successful sperm attraction, capture, and penetration^52^. In our study, the expression of both *PTGER2* and *PTGER4* was higher in PCOS GV CCs and enhanced the proliferation of CCs. *HSDL2, CYP11A1, CYP1B1*, and *DHCR24* are involved in the production of testosterone in granulosa cells, and upregulation of these enhances testosterone production^53^. In our study, the expression of these genes was higher in PCOS GV CCs and downregulated in MI and MII, which indicates that testosterone was overproduced mainly at primary follicles.

Genes associated with folliculogenesis are also differentially expressed in PCOS CCs, including I*MMP2L, NRP1, TGFβ1*, and *IL-6 IMMP2L*^54^. *NRP1*, which enhances follicle assembly, may indicate an oocyte with a positive pregnancy outcome when upregulated in CCs^55^. The TGFβ family, which participates in many processes including folliculogenesis and oocyte maturation, is differentially expressed in PCOS patients compared with non-PCOS patients^56^,^57^,^58^. We also observed that TGF-β1 was upregulated in PCOS CCs. IL-6 is secreted by CCs and promotes COC expansion^59^.

Notch family members play a role in a variety of developmental processes by controlling cell proliferation, differentiation, and apoptosis, as well as interaction between physically adjacent cells^60^. In sheep, overexpression of *NOTCH1–2–3* in granular cells and overexpression of its ligand *JAG1* in oocytes may influence oocyte apoptosis^61^. Our study showed that *NOTCH2* was significantly overexpressed in PCOS CCs at different stages, which may suggest that an irregular connection existed between CCs and oocytes in PCOS and influenced oocyte maturation. The *GJA5* encoded protein is a component of the gap junction pathway. Activation of *S1PR1* induces cell-cell adhesion. At the GV stage, these genes were upregulated in PCOS CCs in order to implement atretic oocytes, because gap junction genes in PCOS oocytes were downregulated. *SMO* is member of the sonic hedgehog pathway and promotes the maturation of COCs by enhancing communication between oocytes and CCs^62^. In our study, we found that some members of the ITGA family were differentially expressed in PCOS CCs; for example, *ITGA6* was upregulated in the GV stage but downregulated in the MI and MII stages, which could be explained by the disruption in communication between CCs and oocytes or CCs. Overall, we have shown that in PCOS, the interaction between CCs and CCs or between CCs and oocytes was extremely close when CCs proliferated at an early stage.

*FAS* contains a death domain and plays a central role in the regulation of programmed cell death. Interaction of the *FAS* receptor with its ligand accelerates oocyte aging^63^. And *SOD1* expression is increased in CCs from infertile women^64^. In our results, upregulation of *FAS* may have reflected the poor quality of oocytes at the GV stage. *GREM1* was significantly downregulated in CCs from diminished ovarian reserve and may be a biomarker of oocyte quality^65^. In Chinese women with PCOS, oxidative stress increases significantly comparing with non-PCOS women because of hyperandrogegism^66^. Recent research has been showed that infertile women represented systemic oxidative stress after controlled ovarian stimulation for ARTs^67^. *PRDX3* and *NDUFA1* are mitochondrial antioxidants, and previous research suggested that these genes are upregulated in response to oxidative stress and endoplasmic reticulum stress in CCs, which may accelerate aging^68^. Therefore, at the GV stage, PCOS CCs had higher expression of *PRDX3* and *NDUFA1*, which suggests that the CCs had suffered oxidative damage.

To our knowledge, our study is the first time to analyze the transcriptome level of PCOS COCs at the GV, MI, and MII stages via single-cell RNA-seq. Due to the poor quality of oocytes and arrest of follicular development in PCOS patients, subfertility has become a significant problem in this population, and ARTs are considered third-line treatment^69^. However, the effect of ARTs is not yet clear because opinions about it are contradictory. Therefore, we sought to assess the effect of ART in PCOS COCs both at the transcriptome level and via clinical statistics. Our findings indicate that ARTs not only can induce ovulation in PCOS patients but also repair the poor quality of oocytes at the transcriptome level and increase the ratio of fertilization and cleavage and the quality of embryos. Moreover, our work revealed several causes of subfertility in PCOS patients at an early stage. Firstly, the development of POCS oocytes is delayed because of lower expression of genes associated with meiosis, gap junction, hormone receptors signaling, and secreted factors. Secondly, higher expression of genes associated with DNA repair in PCOS oocytes is the response to the poor environment for oogenesis and could also reflect the inferior quality of PCOS oocytes relative to control oocytes at an early stage. Thirdly, we identified genes associated with cell-cell communication, proliferation, hormone receptor signaling, follicullogenesis, and oxidative stress in PCOS CCs. Finally, the PI3K-Akt signaling pathway, gap junction pathway, calcium signaling pathway, oocyte meiosis pathway, MAPK signaling pathway, steroid hormone biosynthesis pathway, TGF-β signaling pathway, and VEGF signaling pathway may play a vital role in the development of COCs, because most of the differentially expressed genes we identified were involved in these pathways. In conclusion, ARTs can be an effective treatment for subfertile patients with PCOS.

## Materials and Methods

We recruited 42 women with PCOS and 42 women without PCOS, all of whom were undergoing gonadotropin therapy at Anhui First People’s Hospital in Anhui, China. We have recorded several clinical outcomes of these participants and then compared rates of outcomes between with and without PCOS women. Among them, 9 women with PCOS and 7 women without PCOS were willing to donate COCs for research. PCOS patients were confirmed to have at least two of three Rotterdam 2003 criteria for diagnosing PCOS: hyperandrogenism, oligoovulation and/or anovulation, and polycystic ovaries^70^. All control patients had regular menstrual cycles, normal luteal serum progesterone levels, and normal ovarian morphology. We excluded patients with Cushing’s syndrome, congenital adrenal hyperplasia, and androgen-secreting tumors. Spouses of participants were no infertile problems. Stimulation protocol for all the patients was according to the standard long protocol. Patients’ demographic and clinical characteristics such as age, BMI, LH, FSH and rate of oocytes fertilization, cleavage and embryo were also collected. The study protocol was approved by the Research Ethics committee of Anhui First People’s Hospital (No: 2014008) and conducted in accordance with approved institutional guidelines. All participants gave written informed consent.

### Oocyte Retrieval and Isolation of CCs

Ovarian stimulation and oocyte retrieval protocols were carried out based on the Rotterdam European Society for Human Reproduction/American Society of Reproductive Medicine-Sponsored PCOS Consensus^71^. COCs were isolated via ultrasound-guided vaginal puncture and categorized according to the oocyte nuclear maturation stage: GV, MI, or MII.

We collected 11 oocytes from non-PCOS patients and 9 oocytes from women with PCOS (Table 3). About 10 CCs were isolated from each oocyte. A total of 28 CCs groups were isolated from the oocytes with PCOS and with non-PCOS. Based on the Smart-seq2 protocol, each oocyte or about 10 CCs picked up randomly were transferred to a tube with lysis buffer and stored at −80 °C for RNA-seq.

### Library Construction and Sequencing

We referred to the Smart-seq2 protocol for all RNA-seq experiments^72^. Single cells were washed twice with 1XPBS before being placed in lysis buffer. RNA was isolated from single cells and converted into cDNA. Library construction was performed following the Smart-seq2 protocol, and sequenced reads that contained polyA, low quality, and adapters were pre-filtered before mapping. cDNA was sheared into 100- to 150-bp short fragments according to the manufacturer’s instructions. Libraries were pooled and sequenced on Illumina HiSeq2500 sequencers. Data normalization was carried out by transforming mapped transcript reads to fragments per kilobase of transcript per million mapped reads (RPKM). Genes with fragments per kilobase of transcript per million mapped reads (FPKM) >0.5 were retained for analysis.

### Gene Expression Analysis and Statistics

Genes under threshold of *P*adj < 0.05 were called differentially expressed between samples. *P*adj was calculated via a Benjamini-Hochberg method to exclude false positive results. All clinic parameters were expressed as mean values ± standard deviation (SD). Pathway enrichment analysis was performed using the DAVID web tool^73^. When it was showed *P*adj < 0.05, it can be considered as significant pathway. Heatmap was constructed using heatmap.2 function (gplots package) of R. To identify differentially expressed genes in different stages, we used R package gplots o analyze expression levels of selected genes in each group. Clinical outcomes between PCOS group and control group were tested by t-test and Mann-Whitney U test in the software SPSS20.0. Data of age, FSH, androstenedione and percent of high-quality embryo were normal distribution (Shapiro-Wilk, Sig. > 0.05; Levene’s Test, Sig. > 0.05), so t-test of independent sampler was used to analyze the scores of those groups. Because the data of BMI, SBP, DPB, LH, LH/FSH, SHBG, total gonadotropin dose, testosterone, insulin, ovarian volume total (right and left), number of aspirated oocyte, percent of mature oocyte, percent of oosperm, and percent of cleavage were not normal distribution (Shapiro-Wilk, Sig. < 0.05 or Levene’s Test, Sig. < 0.05), we used Mann-Whitney U test to analyze the scores of those groups.

## Additional Information

**How to cite this article**: Liu, Q. *et al*. Single-cell analysis of differences in transcriptomic profiles of oocytes and cumulus cells at GV, MI, MII stages from PCOS patients. *Sci. Rep.* **6**, 39638; doi: 10.1038/srep39638 (2016).

**Publisher's note:** Springer Nature remains neutral with regard to jurisdictional claims in published maps and institutional affiliations.

## Figures and Tables

**Table 1:** Clinical characteristics and outcome of patients with and without PCOS.

	Non-PCOS (n = 42, number of patients)	PCOS (n = 42, number of patients)	P-value
Age (years)	28.5 ± 3.75	27.4 ± 3.02	0.059
BMI (Kg/m^2^)	22.7 ± 3.1	22.7 ± 3.9	0.929
SBP (mmHg)	113.0 ± 8.4	115.3 ± 9.1	0.148
DBP (mmHg)	74.8 ± 6.5	75.3 ± 8.1	0.830
AFC (antral follicle)	13 ± 5	>24	<0.001 (0.000)^**^
FSH (mlU/ml)	6.7 ± 1.7	6.3 ± 1.3	0.254
LH (mIU/ml)	4.8 ± 2.5	9.3 ± 6.1	<0.001 (0.000)^**^
LH/FSH	0.72 ± 0.3	1.4 ± 0.9	<0.001 (0.000)^**^
Insulin	10.6 ± 4.5 (n = 30)	15.1 ± 11.8 (n = 30)	0.143
Total gonadotropin dose	2175.0 ± 699.3	2068 ± 1035.8	0.580
Testosterone (nmol/L)	1.1 ± 0.6 (n = 40)	1.4 ± 0.7 (n = 40)	<0.05 (0.01)^*^
androstenedione	2.3 ± 0.8 (n = 29)	2.2 ± 0.7 (n=29)	0.931
SHBG (nmol/L)	44.8 ± 19.9 (n = 28)	49.7 ± 34.6 (n = 28)	0.519
oligoamenorrhoea	0	12	<0.001 (0.000)^**^
Ovarian volume total	3737.0 ± 1863.7	5475.9 ± 1800.8	<0.001 (0.000)**
Right	2053.4 ± 1189.2	2964.9 ± 1081.9	<0.001 (0.000)^**^
Left	1683.6 ± 899.7	2511.0 ± 950.0	<0.001 (0.000)^**^
No. of aspirated oocytes	14	20	<0.001 (0.007)^**^
% of mature oocyte (MII)	84.8 ± 19.6	90.1 ± 15.2	0.182
% of oosperm	71.4 ± 21.7	80.1 ± 17.2	<0.001 (0.04)^**^
% of cleavage	70.0 ± 21.7	79.4 ± 18.6	<0.001 (0.033)^**^
% of high-quality embryo	31.2 ± 17.0	37.9 ± 16.8	0.07

*P < 0.05, **P < 0.001, non-PCOS VS PCOS group. BMI, Body Mass Index. SBP, Systolic Blood Pressure. DBP, Diastolic Blood Pressure. FSH, Follicle Stimulating Hormone. LH, Luteinizing Hormone. SHBG, Sex Hormone Binding Globulin.

**Table 2:** Numbers of differentially expressed genes in oocyte and CCs from PCOS patients comparing with control at different stages.

	GV	MI	MII
**Oocyte**	Class I	Class II	Class III
Up	156	184	487
Down	450	253	352
**Cumulus cells**	Class IV	Class V	Class VI
Up	4371	1587	2450
Down	985	780	1504

*P*adj < 0.05, Class I, PCOS oocytes versus normal oocytes at the GV stage), class II, PCOS oocytes versus normal oocytes at the MI stage; class III, PCOS oocytes versus normal oocytes at the MII stage; class IV, PCOS CCs versus normal CCs at the GV stage; class V, PCOS CCs versus normal CCs at the MI stage; class VI, PCOS CCs versus normal CCs at the MII stage.

**Table 3:** Numbers of oocyte and cumulus cells at different stages analyzed by single-cell RNA-sequencing.

	GV (oocyte/CC)	MI (oocyte/CC)	MII (oocyte/CC)
Normal	3/3	4/5	4/6
PCOS	3/4	3/6	3/4
